# Changing Autonomy in Operative Experience Through UK General Surgery Training: A National Cohort Study

**DOI:** 10.1097/SLA.0000000000003032

**Published:** 2018-09-27

**Authors:** Elizabeth J. Elsey, Gareth Griffiths, Joe West, David J. Humes

**Affiliations:** ∗ Division of Epidemiology and Public Health, School of Medicine, University of Nottingham, Clinical Sciences Building 2, City Hospital, Nottingham, UK; † Ninewells Hospital, Dundee, Scotland, UK; ‡ NIHR Nottingham Biomedical Research Centre, Nottingham University Hospitals NHS Trust, Nottingham, UK; § Nottingham Digestive Diseases Centre, School of Medicine, University of Nottingham, Nottingham, UK.

**Keywords:** competency-based education, general surgery, operative competency, operative experience, training and education

## Abstract

**Objectives:**

To determine the operative experience of UK general surgery trainees and assess the changing procedural supervision and acquisition of competency assessments through the course of training.

**Background:**

Competency assessment is changing with concepts of trainee autonomy decisions (termed entrustment decisions) being introduced to surgical training.

**Methods:**

Data from the Intercollegiate Surgical Curriculum Programme and the eLogbook databases for all UK General Surgery trainees registered from August 1, 2007 who had completed training were used. Total and index procedures (IP) were counted and variation by year of training assessed. Recorded supervision codes and competency assessment outcomes for IPs were assessed by year of training.

**Results:**

We identified 311 trainees with complete data. Appendicectomy was the most frequently undertaken IP during first year of training [mean procedures (mp) = 26] and emergency laparotomy during final year of training (mp = 27). The proportion of all IPs recorded as unsupervised increased through training (*P* < 0.05) and varied between IPs with 91.2% of appendicectomies (mp = 20), 40.6% of emergency laparotomies (mp = 27), and 17.4% of segmental colectomies (mp = 15) recorded as unsupervised during the final year of training. Acquisition of competency assessments increased through training and varied by IP.

**Conclusions:**

The changing autonomy of trainees through the course of an entire training scheme, alongside formal competency assessments, may provide evidence of changing entrustment decisions made by trainers for different key procedures. Other countries utilizing electronic logbooks could adopt similar techniques to further understanding of competency attainment amongst their surgical trainees.

## Abstract 2

Supplemental Digital Content is available in the text

Decreasing operative experience during surgical training is a contentious issue internationally.^1–7^ A recent systematic review demonstrated wide variation in the operative experiences of general surgery trainees worldwide.^8^ It has, however, been recognized that simply undertaking a minimum number of procedures does not necessarily confer competency.^8–10^ Assessing competency in surgical training and, particularly, operative procedures is complex.^11^ In the wider context of competency-based medical education, there is an international trend toward moving away from individual case assessments to broader, more generalizable assessments of a trainee's ability to manage defined tasks of work.^12–17^ An “Entrustable Professional Activity” is defined as a unit of activity undertaken which the trainee could be trusted to complete.^18^ ten Cate established a scale to describe the degree to which a trainee could be trusted to perform that unit of activity independently.^19^ This scale closely relates to the way in which the supervision level of an operation is coded in the UK^20,21^ (Table 1).

**TABLE 1:** Entrustable Professional Activity Levels and Supervision Coding for Operative Procedures in the UK

ten Cate Entrustable Professional Activity Supervision Levels^19^	Supervision Coding for Operative Procedures in the UK (adapted)^20^
Observation but no execution, even with direct supervision	Assisting: the trainer completes the procedure from start to finish
Execution with direct, proactive supervision	Supervised, trainer scrubbed: the trainee performs key components of the procedure with the trainer scrubbed
Execution with reactive supervision, ie, on request and quickly available	Supervised, trainer unscrubbed: the trainee completes the procedure from start to finish. The trainer is unscrubbed and is in the operating theater throughout or in the operating theater suite and regularly enters the operating theater during the procedure.
Supervision at a distance and/or post hoc	Performed: The trainee completes the procedure from start to finish. The trainer is not present in the operating theater.
Supervision provided by the trainee to more junior colleague	Training a more junior trainee: a non-consultant grade surgeon training a junior trainee.

Entrustment decisions (a decision by a trainer to allow a trainee to complete a unit of work independently) have further been described as either ad hoc or summative decisions.^22^ Ad hoc entrustment decisions are described as based upon estimated ability of the trainee, potential risk to the patient and contextual issues such as time of day, urgency, and staff availability. These decisions typically take place daily in clinical practice. In contrast, summative entrustment decisions are more formal and made deliberately based upon an established relationship between trainee and trainer with sufficient opportunity for trainee capability evaluation.^22^ In line with this model of entrustment, it could be reasonable to infer that a trainee who is trusted to perform a procedure independently (whether in an ad hoc or more formal decision-making process) has been deemed competent, by their trainer.

General surgery specialty training in the UK is a 6-year program, following a minimum of 4 years postgraduate experience as a Foundation and Core trainee (2 yr each). Trainees follow a curriculum,^23^ which is compulsory and standardized across the UK. The curriculum includes a syllabus of knowledge, clinical skills, and procedural skills to be fulfilled by completion of training.^24^ The Intercollegiate Surgical Curriculum Programme (ISCP) is an online surgical training management system, which hosts the curriculum and also includes an online portfolio of training evidence including work-based assessments, records of supervision meetings, and annual review outcomes. The training program is typically arranged into 6-month placements in a variety of general surgery posts at both large university hospitals and smaller district hospitals. Trainees are supervised through a formal process with named Clinical Supervisors (responsible for day-to-day supervision), an Assigned Educational Supervisor (responsible for reviewing a trainee's progress throughout placements) and a Training Programme Director (a lead for surgical training who oversees all trainees in a geographical region).^25,26^ In addition to regular supervisor meetings, trainee competency progression is assessed annually by a panel comprising regional trainers, external regional representation, lay representation and usually chaired by the Training Programme Director^25^ (Supplementary information online).

In the UK, operative competency is assessed using a work-based assessment tool termed the Procedure-Based Assessment (PBA).^27^ The PBA is designed for both formative and summative assessment purposes (when used in combination with other evidence, such as supervisory reports and logbook records) with a criterion-referenced global rating scale (0–4) that describes trainee competency. A level 4 outcome is described as “Competent to perform the procedure unsupervised (could deal with any complications that arose).” All trainers receive training in how to use the PBA tool. Trainees and trainers identify cases for assessment before commencing the procedure and the trainer is present throughout the procedure to assess the trainee's competency. Outcomes and feedback are recorded in the trainee's electronic portfolio (the ISCP database). In addition to assessments of procedural skill, trainees are expected to undertake minimum indicative numbers of operations, both in total and for 6-key procedures (80 appendicectomies, 50 cholecystectomies, 60 inguinal hernia repairs, 100 emergency laparotomies, 20 segmental colectomies, 5 Hartmann procedures).^28^ As in other systems around the world (eg, USA and Australasia), data are recorded relating to trainee operative experience using a single, approved online logbook.^29–31^ Data are collected for each trainee relating to operative experience in terms of number and type of procedures undertaken and trainee role in each procedure.^32,33^ Trainee role is classified depending on the degree of involvement in the case with clear published descriptors.^20,31,34^

Although data recorded relating to operative experience in surgical training have previously been reported, it has only been used to provide evidence solely related to attainment of procedural numbers.^1,7,35^ Increasing trainee procedural competency is likely to be directly related to trainees being trusted to perform procedures with decreasing supervision. This has, however, not been shown empirically. It may be possible to use existing, routinely collected operative experience data to observe and quantify this relationship. Assessing the types of procedures undertaken over time may also add evidence that a trainee is progressing to more complex operating. Assessment of operative experience data, with analysis of trainee progression through the supervision scale, alongside formal competency assessments for procedures, could demonstrate evidence of increasing trainer entrustment and the ability of a trainee to work with increasing autonomy performing more complex procedures.

The aim of this study was to determine how many operations (both in total and for key procedures) trainees undertake in general surgery training and how the supervision of these changes over the course of training. A secondary aim was to assess whether routinely collected surgical training data from the UK could be used to provide additional evidence for the competency progression of an entire cohort of trainees with a reflection of entrustment decisions through the course of a training scheme.

## METHODS

### Data Sources

This study used routinely collected UK-wide surgical training data held by the ISCP database,^23^ the Joint Committee for Surgical Training (JCST) Surgeons Information Management System (SIMS) database, the Joint Committee in Intercollegiate Examinations database^36^ and the eLogbook database.^29^ All databases used are mandatory repositories of surgical training data in the UK.

The ISCP database was used to define training dates (start of training date, training stage start and end dates, and the predicted or actual date for completion of training. The ISCP database was also used to define trainee status in training (whether the trainee had completed training or remained in training) and for records of PBAs. Data relating to assessments included date of assessment, type of assessment, and the global rating score. The JCST SIMS database was used to define any Out of Programme (OOP) periods taken and the type and duration of OOP (eg, Out of Programme for Research, Out of Programme for Training). All general surgery trainees are required to pass the Fellowship of the Intercollegiate Royal Colleges of Surgery examination to complete training.^36^ At the time of examination, trainees declare a special interest which is recorded in the Joint Committee in Intercollegiate Examinations database. The eLogbook is an online personal registry of operative experience^29^ that contains data including the type of procedure undertaken, the National Confidential Enquiry into Peri-Operative Deaths urgency classification^37^ and the supervision level the operation was carried out under.^20^ Trainee operative logbooks are reviewed regularly by the clinical and educational supervisory team in addition to review at the trainee's Annual Review of Competency Progression; an annual, compulsory review of the trainee's progress in training.^25^

Data from all databases were extracted by the relevant data managers. The data were then linked using the unique identifier (GMC) number and anonymized by the ISCP data manager. All subsequent data management and analysis were performed using Stata 14 (Statacorp, TX).

### Study Population and Timeline

General surgery trainees registered with the ISCP between August 1, 2007 and June 1, 2016 and who had completed training were included. Start of specialty training dates were assessed in both the ISCP and JCST SIMS databases and adjusted accordingly in cases in which dates which did not correspond to the start of specialty training. Trainees who had not completed training were excluded. Trainees who did not have complete stage of training dates (start and end date for each level of training) were excluded. The start date of the study period was the start date of the trainee's specialty training and the end of the study period was the date that the trainee was recommended for completion of training. Any periods of training recorded as OOP which do not count toward training (any OOP period other than Out of Programme for Training) were excluded from the study period in accordance with the Gold Guide for Postgraduate Specialty Training.^25^

The special interests of endocrine, general surgery, and transplant were combined to prevent reporting of 5 or fewer trainees. Oesophagogastric interest and hepatopancreaticobiliary special interests were combined with those declaring upper gastrointestinal (UGI) surgery and reported as a single group of UGI special interest trainees.

### Operative Experience Analysis

Any procedures recorded in the eLogbook which do not count toward total operative numbers required for completion of training in the JCST guidance were excluded from the study (eg, flexible sigmoidoscopy).^28^

Supervision levels were coded to reflect the supervision levels described by JCST [Assisting (A); Supervised, Trainer Scrubbed (STS); Supervised, Trainer Unscrubbed (STU); Performed (P); Training a more junior trainee (T)].^20^ Any procedures coded as observed were excluded. Procedures coded as “Teaching a more junior trainee” were included under the supervision level “Performed” for the purposes of counting procedures undertaken unsupervised. Any operations recorded in the trainee's eLogbook undertaken outside of the study period or whilst the trainee was on an OOP period not contributing toward training were excluded. Any procedures recorded as undertaken in the private sector were excluded.

#### Total Operative Experience

Total operative experience was calculated for each trainee, including procedures recorded as assisting.^28^

#### Index Procedure Experience

Index procedure (IP) experience was calculated in accordance with the algorithms used to calculate IP summary data for the purposes of Annual Review of Competency Progression. Variation in number and type of IP undertaken by sex, region, and stage of training were assessed. Variation in supervision level recorded by stage of training for each IP was assessed.

#### Procedural Competency Assessment Outcomes

Trainee PBA outcomes were assessed and the trainee's stage of training was determined corresponding to the date of their first recorded level 4 PBA. The proportion of trainees awarded their first level 4 PBA was calculated for each stage of training. It was not possible to include emergency laparotomy due to the heterogeneity of procedures included within this umbrella term.

### Analysis

Basic demographics were calculated for the included trainees and quantified using summary statistics. Variation by sex, region, special interest, and supervision level were assessed using simple summary statistics and appropriate parametric and nonparametric tests (*T*-test andχ^2^). Kruskal-Wallis test was used to test for variance in IPs between special interests (non-normally distributed data). Statistical significance was taken at *P* < 0.05.

## RESULTS

### Cohort Demographics

Of the trainees in the dataset, 360 trainees had completed training. A further 49 trainees were excluded from the analysis as they did not have complete stage of training dates leaving a total of 311 trainees included in the study.

Of the 311 trainees included in the study, 243 trainees (78.1%) were men and 68 trainees (21.9%) were women. The median age at start of training was 30.7 years [interquartile range (IQR) 29.3–32.9 yr]. The total time in training (excluding OOP periods not counted toward training) was a median 6.0 years (IQR 6.0–6.5 yr). Colorectal surgery was the most commonly declared special interest (40.5% of the cohort, n = 126) (Table 2).

**TABLE 2:** Demographics of Study Cohort

Demographics of included trainees (n = 311)	
Age at start of training, median (IQR)	30.7 (29.3–32.9)
Sex n (%)	
Males	243 (78.1)
Females	68 (21.9)
Time in Training (adjusted) median years (IQR)	6.0 (6.0–6.5)
Special interest n (%)	
Colorectal	126 (40.5)
Upper gastrointestinal	76 (24.4)
Breast	45 (14.5)
Vascular	43 (13.8)
General/transplant/endocrine	21 (6.8)

### Total Operative Experience

The mean total operative experience for the cohort was 2060 [standard deviation (SD) = 535] procedures at completion of training. There was no significant difference in the mean total operations undertaken between sexes (*P* = 0.29). Of the total operations recorded per trainee, a mean 493 (SD = 212) operations were recorded as Assisted, 602 (SD = 182) as Supervised, Trainer Scrubbed, 198 (SD = 153) as Supervised, Trainer Unscrubbed, and 768 (SD = 282) as Performed or Training a more junior trainee. There was no significant difference observed between sexes in the total numbers of operations recorded at each supervision level.

Variation in total operative experience between special interest groups was seen with a range of means between 1865 (SD = 619) procedures in the vascular trainee group and 2143 (SD = 545) procedures in the colorectal trainee group (*P* = 0.035).

### Index Procedure Experience

Trainees completing training had undertaken a mean 148 appendicectomies (SD = 56), 117 inguinal hernia repairs (SD = 44), 175 cholecystectomies (SD = 90), 82 segmental colectomies (SD = 48), 14 Hartmann procedures (SD = 8), and 114 emergency laparotomies (SD = 42).

**FIGURE 1:**
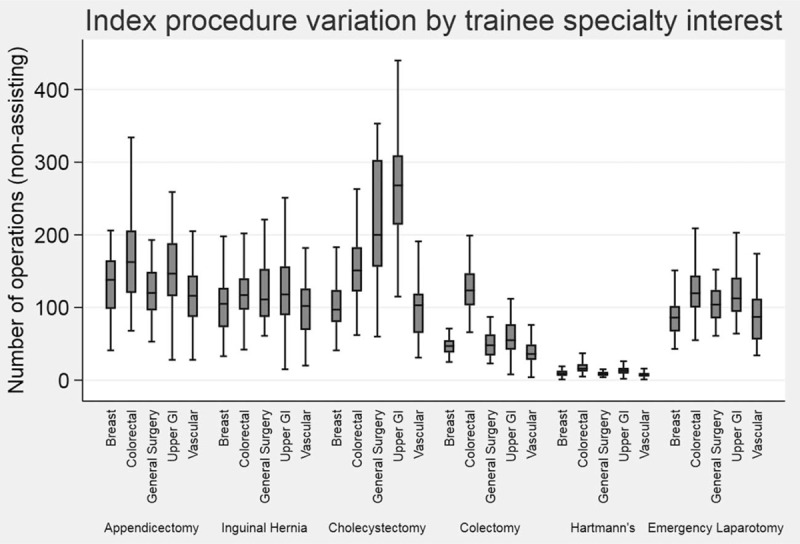
Variation in the mean number of non-assisting index procedures at completion of training by special interest.

Wide variation was found between special interest groups for all IPs undertaken (*P* < 0.0001 across all IPs) (Fig. 1). Analysis of variance showed that the association between special interest and number of segmental colectomies undertaken was statistically significant with colorectal trainees undertaking a median 124 segmental colectomies (IQR 103–147) compared with a median 36 segmental colectomies (IQR 28–49) undertaken by vascular trainees (*P* < 0.0001). UGI trainees undertook the most cholecystectomies with a median 268 procedures (IQR 214–310) compared with a median 103 cholecystectomies (IQR 65–119) undertaken by vascular trainees (*P* < 0.0001). Breast special interest trainees undertook the fewest emergency laparotomies [median 86 (IQR 67–102)] compared with colorectal trainees who undertook a median 120 (IQR 100–144) emergency laparotomies (*P* < 0.0001).

### Changing Volume of Index Procedures Through Training

Variation in the type of IPs undertaken by training stage was seen (Table 3). The most commonly undertaken IP as primary surgeon (non-assisting supervision codes) during the first year of training was appendicectomy with a mean 24 procedures recorded per trainee. The mean number of non-assisting appendicectomies recorded at each stage of special training remained relatively static reflecting the service provision of emergency general surgery training in the UK. The mean number of non-assisting inguinal hernia procedures recorded decreased from 19 during the first year of training to 10 during the final year of training indicating the simpler case load of early specialty training.

**TABLE 3:** Mean Number of Index Procedures (Non-assisting Supervision Codes) Undertaken per Trainee (n = 311) by Training Year

	Mean Procedures Per Trainee (SD)				
Training Year	Appendicectomy	Inguinal Hernia	Cholecystectomy	Colectomy	Emergency Laparotomy
1	24 (15)	19 (14)	17 (18)	6 (6)	13 (9)
2	25 (15)	19 (14)	23 (18)	9 (8)	16 (10)
3	26 (15)	18 (13)	25 (21)	9 (8)	18 (12)
4	24 (15)	15 (14)	26 (24)	11 (10)	21 (13)
5	23 (19)	12 (13)	22 (26)	12 (16)	23 (16)
6	19 (16)	10 (10)	20 (28)	13 (14)	23 (16)
Total^*^	148 (56)	117 (44)	175 (90)	82 (48)	114 (42)

^*^Mean total procedures undertaken by completion of training.

In contrast, the number of more complex IPs increased in throughout the course of training. The mean number of non-assisting emergency laparotomies undertaken rose throughout training from a mean of 13 procedures recorded during the first year of training to 23 procedures recorded during the final year of training. The mean number of colectomies undertaken as primary surgeon increased from 6 during the first year of training to 13 during the final year of training.

### Changing Supervision of Index Procedures Through Training

**FIGURE 2:**
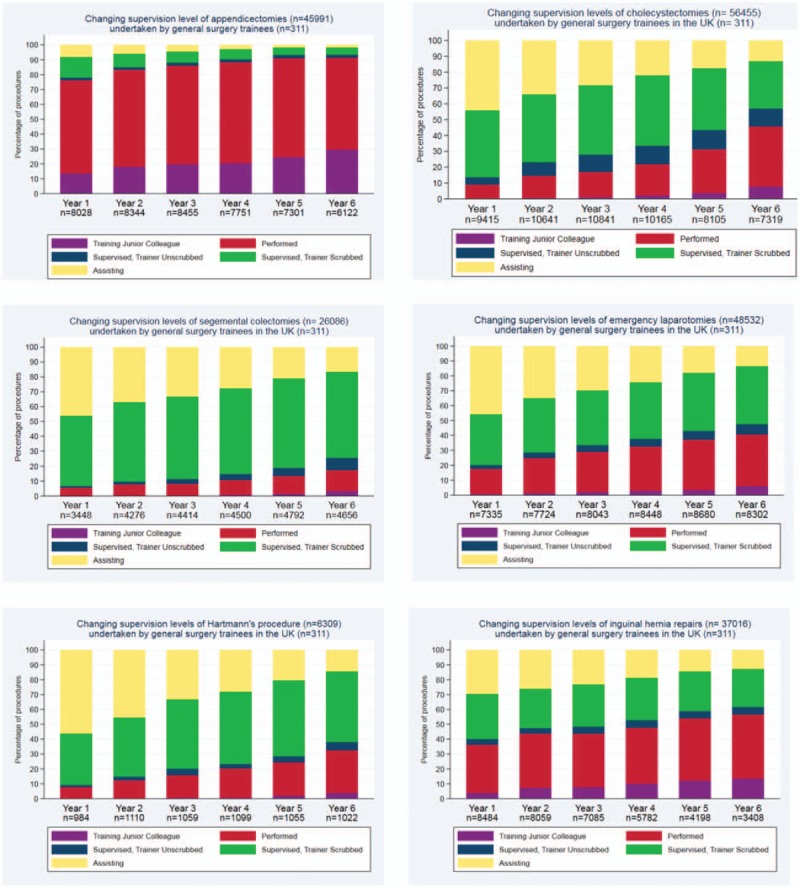
Changing supervision levels for index procedures undertaken by UK general surgery trainees.

The changing supervision levels recorded for each IP are shown in Figure 2. The proportion of IPs recorded as unsupervised (Performed or Teaching a more junior colleague supervision codes) increased for all procedures as trainees progressed through training with a corresponding decrease in the number of procedures recorded as assisting. There was a difference in the pattern of change seen between the different IPs.

Of the appendicectomies recorded during the first year of training, 78.0% were recorded as unsupervised indicating a degree of competency obtained in appendicectomy prior to commencing specialty training. The proportion of appendicectomies recorded as undertaken unsupervised increased to 91.2% during the final year of specialty training with 29.5% recorded as Teaching a more junior trainee. Of the cholecystectomies recorded during the first year of training, 11.9% were recorded as unsupervised, increasing to 47.2% during the final year of training (7.8% Teaching a more junior trainee).

Of the more typically complex operative procedures, a smaller proportion of procedures recorded as unsupervised was observed. During the first year of training, only 5.6% of segmental colectomies were recorded as unsupervised, whereas the proportion of segmental colectomies recorded as unsupervised rose to 17.4% (14.1% Performed, 3.3% Teaching) during the final year of training.

Similarly, the proportion of emergency laparotomies recorded as unsupervised during the first year of training was 17.7% with an increase to 40.6% during the final year of training. For both segmental colectomy and emergency laparotomy, the proportion of procedures recorded as supervised training (Supervised, Trainer Scrubbed or Supervised, Trainer Unscrubbed) during the final year of training were high at 66.1% and 46.0%, respectively, reflecting ongoing supervised training of trainees at a senior level.

### Procedural Competency Assessment

The proportion of trainees awarded a level 4 PBA by completion of training was 85.5% for appendicectomy, 79.7% for colectomy, 89.7% for inguinal hernia, and 91.3% of trainees for cholecystectomy. Low numbers of trainees recorded a level 4 PBA in the early years of specialty training for more complex procedures (9.0% and 18.3% of trainees completing the second year of training for segmental colectomy and cholecystectomy, respectively) with a trend toward obtaining level 4 PBAs later in training (*P* < 0.05). More trainees were awarded level 4 PBAs in early years of training for less complex procedures (36.3% and 40.2% of trainees completing the second year of training for appendicectomy and inguinal hernia repair, respectively) with no significant trend seen for award of level 4 PBA by training stage (*P* = 0.15 appendicectomy; *P* = 0.24 inguinal hernia) (Table 4).

**TABLE 4:** Number of Trainees (Total n = 311) Awarded an L4 Procedure Based Assessment (PBA) in General Surgery Index Procedures by Training Year

	Trainees Achieving L4 PBA^*^			
Training Year	Appendicectomy	Inguinal Hernia	Cholecystectomy	Segmental Colectomy
1	48 (15.4%)	43 (13.8%)	10 (3.2%)	7 (2.3%)
2	113 (36.3%)	125 (40.2%)	57 (18.3%)	28 (9.0%)
3	170 (54.7%)	193 (62.1%)	116 (37.3%)	67 (19.6%)
4	214 (68.8%)	232 (74.6%)	190 (61.1%)	128 (41.2%)
5	245 (78.8%)	261 (83.9%)	251 (80.7%)	205 (65.9%)
6	266 (85.5%)	279 (89.7%)	284 (91.3%)	248 (79.7%)
*P* value for trend	0.15	0.24	<0.05	<0.05

^*^Cumulative total throughout years of training.

## DISCUSSION

Using data relating to the entire course of training for all trainees in the UK we have been able to map changing operative experience in a new way to provide empirical evidence of changing autonomy in surgical training. This study has described a change in types of procedure undertaken through the course of surgical training, evidencing progression from more simple procedures at the start of training to complex procedures by the completion of training. We have demonstrated that as trainee seniority increases, the level of supervision of their operating decreases but that this trend differs between types of procedure, according to complexity. By using data from a formal assessment tool (PBA) we have also described an increasing proportion of trainees achieving a competency standard, in differing IPs through the course of training. By using routinely collected national surgical training data in this way, we have been able to demonstrate evidence of changing entrustment decisions through the course of an entire training program for a national cohort of trainees.

Previous studies of operative experience in general surgery were limited to describing total operative numbers alone with several studies only using select groups of trainees, single region data or logbook consolidation sheets rather than centrally held, national data.^10,35,38,39^ This study is strengthened by its use of centrally held data from a national training scheme for an entire cohort of trainees. Use of complete training records, validated in real time by both trainees and trainers, along with annual review of individual trainee data, adds to the reliability of the data used. Careful data management to accurately calculate total IPs, exclusion of procedures which do not count toward total operative experience and account of accurate training timelines all increase the reliability of data presented in this study. Inaccuracies in the degree of case supervision recorded by trainees may limit the reflection of independent practice in this study. However, clear definitions of supervision levels are published by the JCST and to overstate involvement in a case would be considered a probity issue with clear, negative consequences.^20^ Furthermore, trainee logbooks are reviewed regularly throughout training placements by supervisors and at annual training reviews, adding a degree of validation to the data. A limitation of the study is acknowledged in the understanding that supervised versus unsupervised operating does not necessarily reflect a trainee's capability to perform a procedure but may merely reflect who was present in theater. The limited availability of an assistant, who is not the trainee's supervising trainer, will inevitably impact on the supervision of case, necessitating a supervision coding of Supervised, Trainer Scrubbed or Supervised, Trainer Unscrubbed rather than entirely unsupervised operating. Furthermore, explicit entrustment decisions taken by trainers for the purposes of summative competency assessment are more formal than the daily ad-hoc supervision decisions made by supervising trainers, which have many other influences including time of day, staff availability, and urgency of the procedure. Although this limits the conclusions that can be drawn regarding entrustment decisions in UK practice from logbook data alone, we have been able to demonstrate the changing autonomy of trainees through training, reflecting increasing trust to perform procedures unsupervised.

The authors acknowledge the apparent anomalies of the small numbers of early specialty trainees recording complex procedures unsupervised (5.6% of colectomies recorded as unsupervised) and achieving competency assessments for independent practice (2.3% of trainees awarded a level 4 PBA outcome) by completion of the first year of specialty training. This is explained by the varying operative experience and competency of trainees commencing specialty training in the UK. Some trainees, with completion of overseas training before entering UK general surgery specialty training, are already technically proficient in a wide range of procedures. Some UK trainees may have completed additional training or service provision years prior to specialty training and are experienced technically. It is likely that such trainees are allowed to undertake more complex procedures sooner, reflected in the small proportion of year 1 and year 2 trainees undertaking colectomies (5.6% in year 1) or emergency laparotomies unsupervised (17.1% in year 1) and achieving level 4 PBAs (2.3% in year 1 and 9% in year 2 for colectomy). The use of the PBA data in this study, demonstrates that a proportion of trainees are judged to be competent to perform more complex procedures, even at early stages of training. The pattern of award of level 4 procedural competency assessment relates to the changing supervision of IPs, adding evidence to the supposition that procedural autonomy decisions appear to mirror entrustment decisions in the UK.

Wagner et al recently published a study of the use of Entrustable Professional Activities in the assessment of operative capabilities, together with the perceived and actual autonomy, of general surgery residents in a single USA center.^16^ Similar to the findings of decreased supervision through training in this study, the authors reported that perceived autonomy was greater in senior trainees than more junior trainees.^16^ The authors reported that approximately 40% of faculty members said that trainees were not independently capable of performing inguinal hernia repair at any stage of training. Comparatively fewer trainees (<20%) reported not being trusted to perform inguinal hernia repairs independently.^16^ In contrast, our study demonstrates that 56.5% of inguinal hernia repairs undertaken by final year trainees in the UK were performed unsupervised and 89.7% of final year trainees had been awarded a level 4 PBA for inguinal hernia repair. The difference perhaps reflects the extended training duration in the UK and thus increased autonomy of trainees.

Our study relates to the work of George et al with regard to trainee autonomy in general surgery training. George et al^40^ undertook a study of 536 general surgery trainees from a selection of US training programs in 2015/16 and assessed trainee readiness for independent practice using a competency assessment tool and compared this with a judgment of trainee autonomy. The authors described increasing autonomy and performance assessment results through training. Only a small proportion of final year trainees, however, demonstrated near independence. They concluded that US general surgery trainees are not ready to independently practice even common core procedures (appendicectomy, inguinal hernia repair, and cholecystectomy) at completion of training. Comparisons between US and UK general surgery operative competency standards are limited by the notably different durations of training programs. Trainees completing both training schemes are, however, free to practice independently with 20% of US general surgery residents entering directly into independent practice at completion of training.^41^ Eighty percent of US general surgery residents undertake a period of postresidency fellowship training,^41^ and 77% of general surgery trainees in the UK^42^ also pursue additional clinical fellowship periods in addition to standard UK training. This suggests that the majority of trainees in both countries feel the need to extend their clinical training before independent practice, whether for additional operative experience or other reasons such as further development of specialty interest or niche skill training.

Although comparison of outcomes between different general surgery training schemes are limited by the variation in training curricula and cultures, the use of routinely collected data as described in this study could be applied internationally to training schemes which electronically record operative experience data. This study demonstrates a new and alternative way of using operative experience data beyond simple counting to add further evidence to competency attainment through the course of training.

## CONCLUSIONS

The changing autonomy of trainees through the course of an entire training scheme for a national cohort, alongside formal summative competency assessments, may provide evidence of changing entrustment decisions made by trainers for different key procedures. Such methods could be used by other countries utilizing electronic logbooks to further understanding of competency acquisition in surgical training.

## Supplementary Material

Supplemental Digital Content
