# Supplementary material for: Changing Autonomy in Operative Experience Through UK General Surgery Training: A National Cohort Study
Source: Ann Surg. 2018 Sep 27;269(3):399–406. doi: 10.1097/SLA.0000000000003032 (PMC6369871; doi:10.1097/SLA.0000000000003032)
Supplement: Supplemental Digital Content [file ansu-269-399-s001.docx]

### General Surgery Training in the UK

#### Governance of surgical training in the UK

There are several governing bodies for surgical training in the UK which are all linked and work together with trainees and surgical trainers. (Figure 1)


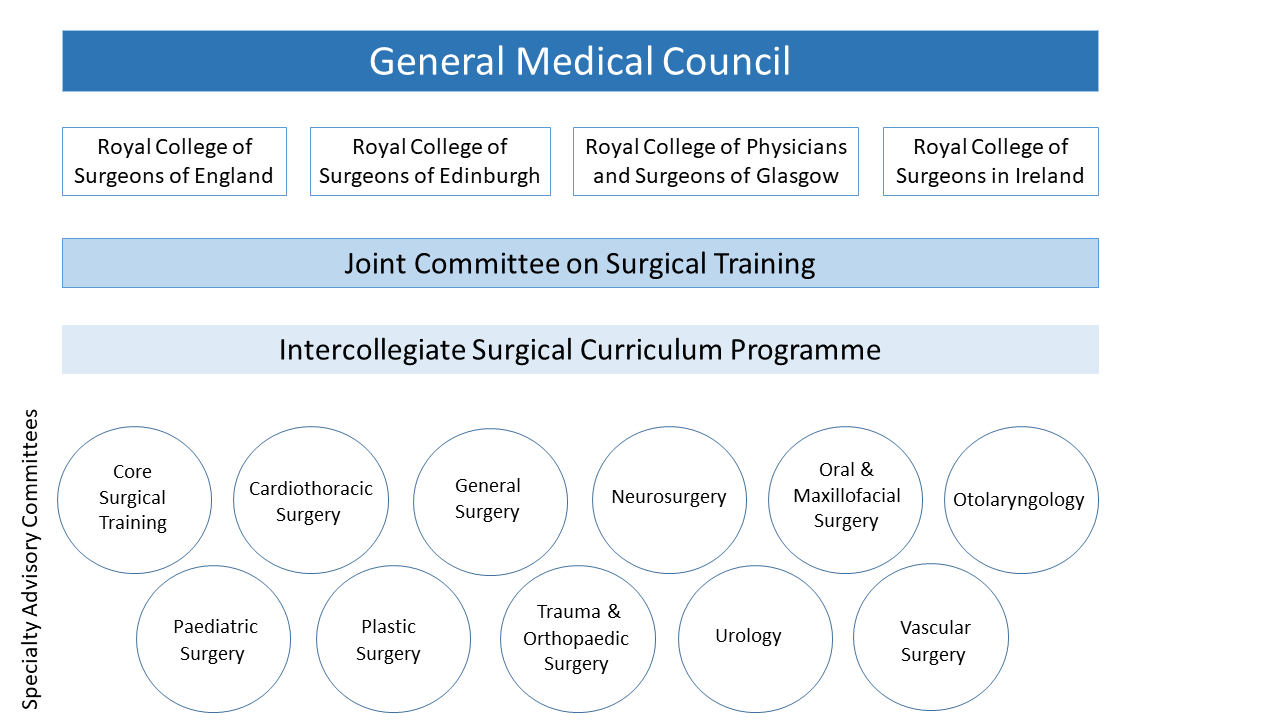


Figure 1 Governing and regulatory bodies for surgical training in the UK

##### General Medical Council

The General Medical Council (GMC) is the UK regulatory body for all medical education and training. They have five main areas of work which are:

1. Maintaining a medical register of doctors approved to practice in the UK

2. Setting standards for doctors including professional values and behaviours.

3. Overseeing medical education and training including approving curricula and monitoring training centres.

4. Ensuring that revalidation processes and standards are met for senior doctors.

5. Investigating and managing concerns about poor medical practice.^1^

##### Royal Colleges of Surgery

There are four Royal Colleges of Surgery:

1. Royal College of Surgeons of England^2^
2. Royal College of Surgeons of Edinburgh^3^
3. Royal College of Physicians and Surgeons of Glasgow^4^
4. Royal College of Surgeons in Ireland^5^

The Colleges are professional membership organisations and provide representation for surgical affiliates, members and fellows in addition providing education and continuing professional development with the aim of advancing standards in surgical care. The Joint Committee on Intercollegiate Examinations (JCIE) are responsible to the four Royal Colleges for the compulsory post- graduate surgical exams required of surgical trainees.

##### Joint Committee on Surgical Training

The Joint Committee on Surgical Training (JCST) which is an intercollegiate advisory body to the four Royal Colleges of Surgery in the UK. The responsibilities of the JCST include the national registry of UK trainees, the recommendation of trainees to the GMC for Certificate of Completion of Training, developing the surgical syllabus and monitoring the quality of surgical training in the UK.^6^ It is also responsible for developing and maintaining the Intercollegiate Surgical Curriculum Programme (ISCP) online training management system. The JCST is the parent body for all the Specialty Advisory Committees (SACs) which are responsible for each surgical specialty.

There are ten surgical specialties in the UK, each with their own SAC:

- Cardiothoracic Surgery
- General Surgery
- Neurosurgery
- Oral and Maxillofacial Surgery
- Otolaryngology
- Paediatric Surgery
- Plastic Surgery
- Trauma & Orthopaedic Surgery
- Urology
- Vascular Surgery

Core Surgical Training is represented by the Core Surgical Training Advisory Committee which also falls under the JCST remit.

##### Intercollegiate Surgical Curriculum Programme

The ISCP was launched in August 2007 to coincide with reforms to post-graduate training. It is a web-based training management system which hosts the surgical curriculum for each surgical specialty, including Core Surgical Training, and has become central to the delivery of surgical training in the UK. It includes a syllabus to be covered during training, criteria to be met for completion of training, online work-based assessments and an online portfolio for evidence of achievement. ^7^ The portfolio includes records of the work-based assessments completed, documents that a trainee has uploaded as evidence of development e.g. conference attendance certification, presentations given to learned societies, letters of thanks from patients and supervisory reports. The evidence in the trainee’s portfolio is used for the trainee’s Annual Review of Competency Progression. Use of the ISCP is compulsory for all surgery trainees.

##### Specialty Advisory Committees

The JCST, through the SACs, have a range of responsibilities including registration of trainees for specialty training, recruitment, logbook development and writing the curriculum to be followed in association with stakeholders. The SACs are also responsible for providing externality to assessment processes e.g. at the trainee’s Annual Review of Competency Progression and for recommending a trainee to the GMC for Certification. The externality and advisory function is provided by the presence of an SAC Liason Member who represents the SAC to a region. The Liason Member must originate from a geographically separate training region in order to provide externality and is a Consultant surgeon.

#### Timeline of post-graduate general surgery training in the UK

Trainees pursuing a career in General Surgery in the UK follow a typical pathway for General Surgical training which has been standardised since 2007.^8^ After graduating from medical school, trainees complete two years of Foundation Training and then two years of Core Surgical Training. Following this, trainees apply for specialty surgical training and if successful, are awarded a National Training Number (NTN) which defines the trainee as being appointed to an approved surgical training programme. General Surgery specialty training is an indicative 6 years in duration. A trainee in the first year of Core Surgical Training is referred to as a Core Trainee, year 1 (CT1) with the second year trainees known as CT2 trainees. A trainee in the first year of specialty training is referred to as a Specialty Trainee, year 3 (ST3). Trainees typically finish standard training at the end of their ST8 year.

Previously, if trainees were unsuccessful at recruitment to specialty training, they could be appointed to Locum Appointment for Training (LAT) posts. LAT posts were typically one year in duration and within an accredited surgical training scheme but were not full training pathway appointments with no progression beyond the end of the fixed-term post. Once a trainee had secured a NTN training appointment, they could apply for retrospective recognition of the time spent in a LAT appointment towards time required for CCT. No LAT appointments have been made in General Surgery in England since 2014 following a move away from these posts. Instead, trainees who fail to obtain a NTN post may apply for Locum Appointment for Service (LAS) posts. Unlike LAT posts, LAS posts are not subject to subsequent approval for recognition towards time in training.

Trainees who have not completed UK approved Foundation and Core Surgical Training posts may also obtain an NTN training appointment for specialty training by demonstrating acquisition of Core Surgical Training competencies and meeting the advertised essential criteria. This is typical for trainees applying for specialty surgery training from overseas. These trainees are awarded a Certificate of Equivalence of Specialty Registration, Combined Programme (CESR CP,) rather than a CCT, at completion of specialty training.^9^ Trainees from overseas who complete UK Core Surgical Training prior to obtaining a NTN training appointment may also be recommended for Certificate of Completion of Training in the usual fashion. In 2011, half of the applicants to General Surgery specialty training were those with a primary medical qualification from overseas and half had primary medical qualifications from the UK. [Personal Communication. Mr Gareth Griffiths, JCST Chair]. The requirements for recommendation for CESR CP are the same as the requirements for recommendation for CCT and both routes allow entry to the GMC Specialty Register enabling independent practice as a Consultant Surgeon in the UK.

The surgical curriculum requires summative demonstration of knowledge attained at two stages in surgical training. The Membership of Royal College of Surgeons (MRCS) is required to complete Core Surgical Training and therefore must be achieved in order to obtain a NTN training appointment. The Intercollegiate Specialty board examination must be passed prior to being awarded CCT and in order to apply for Fellowship of the Royal College of Surgeons (FRCS). It is usually taken during the final two years of specialty training.

Figure 1 shows a typical training pathway with the blue arrow at start of general surgery specialty training and the red arrow indicating the point of completion of training.


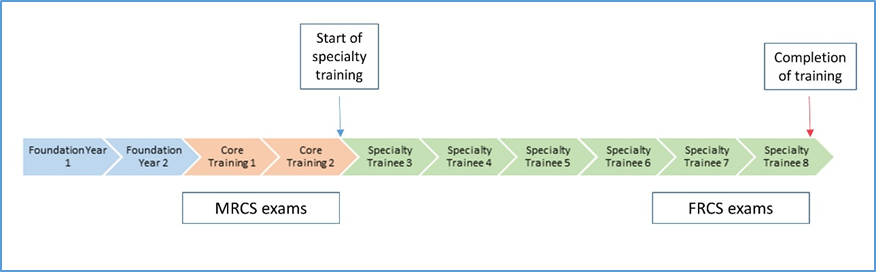


Figure 2 Timeline of postgraduate surgical training in the UK

Trainees may extend their time in training from the standard 6 years for the following reasons:

- sick leave
- maternity leave
- military service leave
- working less than full time
- time out of programme for research (OOPR)
- time out of programme for further training (OOPT)
- time out of programme for other experience (OOPE)
- time out of programme for career break (OOPC)
- extension of training for additional training needs

##### Special Interest

General Surgery trainees are trained in the specialty of General Surgery and, if successful, are recommended for Certification in General Surgery. Trainees must also declare a special interest which reflects the particular area within General Surgery that the trainee wishes to develop their future career in.

Special interests include:

- Colorectal surgery
- Upper Gastrointestinal Intestinal surgery
- Breast surgery
- Endocrine surgery
- Transplant surgery
- Vascular surgery (only permissible for trainees commencing specialty training pre-August 2013)

Trainees may also develop special interests in General surgery of childhood, Advanced Trauma surgery or Remote and Rural surgery alongside one of the above listed special interests. Vascular surgery became an independent surgical specialty with recruitment to Vascular Surgery as a specialty commencing in August 2013. Trainees with NTNs in General Surgery appointed prior to 2013 are still able to declare vascular surgery as a special interest but are unable to transfer their training into the new Vascular Surgery specialty training scheme. General Surgery trainees appointed to NTNs from 2013 are unable to declare vascular surgery as their special interest.

### General Surgery Curriculum

The curriculum is hosted on the ISCP website.^10^ It contains a detailed syllabus with the knowledge, clinical skills and procedural skills expected of trainees by completion of training. It also describes the programme of assessment and the supervision of trainees in the UK alongside details of surgical education, training pathways and quality assurance processes. Trainees are expected to cover the breadth of the curriculum by completing a variety of surgical training placements through the course of the 6-year specialty training. Each training year is typically divided into two 6-month placements. Trainees may remain in the same placement for 12 months but are still required to meet with the CS and AES with the same frequency as those in a 6-month placement. Trainees are placed general surgery posts with a variety of special interests (e.g. colorectal, oesophago-gastric, transplant) with emergency general surgery common to all posts. Placements are at approved regional training centres which vary in size from major, tertiary referral centres affiliated with Universities to smaller district general hospitals. All regions have both tertiary referral centres and district general hospitals to ensure a variety of training environments and case-mix. Each trainee is supervised according to Gold Guide principles including named supervisors with specific training responsibilities and appropriate clinical supervision as set out by standards published by the GMC.^9,11^

#### Training Programme Director

Each region has an appointed Training Programme Director (TPD) for general surgery who is responsible for overseeing the delivery of training regionally. The TPD ensures that training standards set out by the Gold Guide, GMC and JCST are adequately met. Activities include ensuring curriculum delivery and quality of local training, allocating placements, managing trainees in difficulty and overseeing the annual review process. The TPD is Consultant surgeon from the training region but will not necessarily work clinically with most of the trainees they are responsible for.

#### Assigned Educational Supervisor

Each trainee has an Assigned Educational Supervisor (AES) who is responsible for meeting with the trainee at least three times in each 6-month surgical placement. The role of the AES includes setting educational goals with the trainee, creating constructive and career developmental dialogue with the trainee and responsibility for appraisal.^9^ The AES reviews the trainee’s ISCP portfolio and their progress as evidenced by WBAs, logbook review and reports from the Clinical Supervisor.

#### Clinical Supervisor

The Clinical Supervisor (CS) is a named Consultant responsible for the day to day supervision, training and assessment of the trainee. The CS may work in a team of Consultants and share this responsibility with colleagues or may work independently. Trainees have daily contact with their CS when undertaking elective work. It would be expected that a trainee attended all of their CS routine activities such as inpatient operating lists, day-case operating lists, endoscopy lists, out-patient clinics. Additionally, a trainee is responsible for the inpatient care of the CS’s patients and would be expected to communicate with the CS daily regarding clinical updates. The CS provides a written report for the trainee’s portfolio at the end of each placement with a summary of the trainee’s performance and progress. The CS also has access to the trainee’s portfolio including summary reports of the logbook and WBAs.

#### Annual Review of Competency Progression

Progression through surgical training is dependent on the trainee being satisfactorily assessed at a yearly appraisal, termed the Annual Review of Competency Progression (ARCP). The requirements and purposes of the ARCP process are generic to all post-graduate training in the UK.^9^

Trainees are required to provide evidence of meeting curriculum requirements at ARCP e.g achievement of learning goals and satisfactory demonstration of stage-appropriate competencies through completion of WBAs. Trainees are awarded an ARCP outcome (Table 1), with ARCP outcomes 1, 6, 7.1 and 8 deemed to be satisfactory outcomes and the other outcomes reflecting non-standard trainee progression. The ARCP panel is made up of at least 3 members of the regional training committee (regional consultants with CS or AES responsibilities), one of whom is the TPD or more senior member of the regional training board (Head of School of Surgery or Postgraduate Dean). The panel also includes lay representation and an external advisor who is usually the SAC liason member.^9^ The panel makes an independent judgement of the trainee’s online portfolio which includes WBA outcomes, the CS and AES reports and evidence of other activities e.g. courses attended, audit and research documentation.

Table 1 Annual Review of Competency Progression outcomes ^9^

| ARCP Outcome | Definition | Satisfactory/ Unsatisfactory |
| --- | --- | --- |
| 1 | Achieving progress and development of competences at the expected rate | Satisfactory |
| 2 | Development of specific competences required – additional training time not required. | Unsatisfactory |
| 3 | Inadequate progress - additional training time required. | Unsatisfactory |
| 4 | Released from training programme with or without specified competences. | Unsatisfactory |
| 5 | Incomplete evidence presented - additional training time may be required. | Unsatisfactory |
| 6 | Will be recommended as having completed the training programme and for award of a CCT | Satisfactory |
| 7.1 | Satisfactory progress in or completion of the LAT / FTSTA placement. | Satisfactory |
| 7.2 | (Fixed term training) - Development of specific competences required – additional training time not required. | Unsatisfactory |
| 7.3 | (Fixed term training) – Inadequate Progress by the Trainee). | Unsatisfactory |
| 7.4 | (Fixed term training) – Incomplete evidence presented. | Unsatisfactory |
| 8 | Out of programme for research, approved clinical training or a career break (OOPR/OOPT/OOPC). | Satisfactory |

ARCP: Annual Review of competency and Progression. CCT: Certificate for Completion of Training. LAT: Locum appointment for Training. FTSTA: Fixed Term Specialty Training Appointment. OOPR: Out Of Programme Research. OOPT: Out Of Programme Training. OOPC: Out of Programme Career break.

#### Requirements for Certificate of Completion of Training in General Surgery

Trainees must meet minimum requirements in the curriculum prior to being recommended for Certification^12^. These include:

- demonstration of knowledge by passing the FRCS exam
- breadth of experience by completion of required placements in various general surgery specialties
- attainment of specific competencies through WBAs in specified subject areas
- understanding of research evidenced of satisfactory research involvement
- understanding of quality improvement projects evidenced by audit completion
- understanding of medical education principles evidenced by appropriate courses or qualifications
- experience of leadership and management
- trauma care provider certification

Additionally, the curriculum sets out expected indicative operative experience requirements and operative competency requirements.^12^ In order to be recommended to the GMC for award of Certificate of Completion of Training, trainees are judged on a combination of supervisory reports, work-place based assessments including specific requirements for procedural competency and 360-degree appraisals, operative experience records and evidence of having met the above requirements.

#### Requirements for operative experience

All General Surgery trainees are required to meet indicative numerical operative experience requirements for key index procedures and for total operative experience, regardless of special interest.

The index procedures are commonly performed general surgical procedures. The requirements are:

- 80 appendicectomy
- 60 inguinal hernia
- 50 cholecystectomy
- 100 emergency laparotomy to include 20 segmental colectomy and 5 Hartmann’s procedures^10^

These numbers must relate to the trainee having been the primary surgeon in the case. This is defined as non-assisting supervision codes.^13^ The supervision codes for operations in the UK are described as:^13^

**Assisting:** The trainer completes the procedure from start to finish. The trainee performs the approach and closure of the wound. The trainer performs the key components of the procedure

**Supervised, Trainer Scrubbed:** The trainee performs key components of the procedure (as defined in the relevant PBA) with the trainer scrubbed.

**Supervised, Trainer Unscrubbed:** The trainee completes the procedure from start to finish. The trainer is unscrubbed and is:

- in the operating theatre throughout

- in the operating theatre suite and regularly enters the operating theatre during the procedure (70% of the duration of the procedure)

**Performed:** The trainee completes the procedure from start to finish. The trainer is present for <70% of the duration of the procedure. The trainer is not in the operating theatre and is:

- scrubbed in the adjacent operating theatre

- not in the operating suite but is in the hospital

**Training a more junior trainee:** A non-consultant grade surgeon training a junior trainee.

Additionally, trainees have to perform a total of 1600 procedures through the course of their specialist general surgical training. This total value is inclusive of cases where the trainee had an assisting role.^12^ Operations are recorded in the eLogbook by trainees and reviewed at clinical supervision meetings, educational supervision meetings and at ARCP. The eLogbook is a single, secure, standardised, electronic database used for recording operative experience across all of specialty surgery training. It was linked to ISCP portfolios in 2009 with data linked between the two databases by the trainee’s GMC number. Use of the eLogbook is compulsory and summaries can be produced indicating the types , quantities and supervision of procedures that trainees have recorded over define time periods.^14^

These numerical requirements were introduced in the 2013 iteration of the General Surgery curriculum following a consensus decision of UK surgical training leads and a study of general surgery trainee logbooks. The numbers were based on the 25^th^ percentile of operative experience recorded in the assessed logbooks.^15^

#### Requirements for operative competence

Trainees must complete work-based assessments and attain assessment levels described in the completion of training certification guidelines in order to demonstrate operative competence.^12^ By the end of training, general surgeons are expected to have achieved three Procedure Based Assessments (PBA) at level 4, judged by three separate assessors, in the same index procedures described for operative experience: appendicectomy, inguinal hernia, cholecystectomy, emergency laparotomy, segmental colectomy, Hartmann's.^12^

The indicative numbers and PBA requirement standards were introduced in the 2013 version of the curriculum. These standards were flexibly applied to trainees who were appointed to specialty training prior to 2013 depending on their stage of training and at TPD discretion.

##### Procedure Based Assessment

In the UK, the work-based assessment tool used to assess operative competency is the Procedure-Based Assessment (PBA). The PBA model of assessment is designed to assess the “does” element of Miller’s clinical competence model.^16^ The PBA tool is made up of a series of binary, criterion referenced ratings for task specific points as well as a global summary ordered categorical assessment (rated 0-4) of the trainee’s ability to perform the procedure.

The possible global summary scores for a PBA are:

**Level 0:** Insufficient evidence observed to support a summary judgement

**Level 1:** Unable to perform the procedure, or part observed, under supervision

**Level 2:** Able to perform the procedure, or part observed, under supervision

**Level 3:** Able to perform the procedure with minimum supervision (needed occasional help)

**Level 4:** Competent to perform the procedure unsupervised (could deal with any complications that arose)^17^

Each PBA is procedure specific and has task specific criteria written to reflect that particular operation. This is designed to be used to guide formative feedback for particular elements of the procedure that could be improved upon. The global rating score is generic to all PBAs. The global summary score is used for both formative and summative purposes. Assessment guidance is published on the ISCP website and suggests that the assessment should be completed immediately after the procedure with both the trainer (assessor) and trainee present for feedback purposes. All surgical trainers are required to be trained in the use of WBAs (either face to face, web-based or written guidance.) The PBA was assessed by the National Institute of Health Research (NIHR) Health Technology Assessment programme and shown to be a valid and reliable assessment tool.^17^ It has been a formal component of the surgical training assessment programme since 2007 and is now a well-established tool in the UK.^18^

1. General Medical Council Homepage. <https://www.gmc-uk.org/>. Accessed 13th June, 2018.

2. The Royal College of Surgeons of England Homepage. <https://www.rcseng.ac.uk/>. Accessed 18th June, 2018.

3. The Royal College of Surgeons of Edinburgh Homepage. <https://www.rcseng.ac.uk/>. Accessed 18th June, 2018.

4. The Royal College of Physicians and Surgeons of Glasgow Homepage. <https://rcpsg.ac.uk/>. Accessed 18th June, 2018.

5. The Royal College of Surgeons in Ireland Homepage. <https://www.rcsi.com/dublin/>. Accessed 18th June, 2018.

6. Joint Committee for Surgical Training Homepage. <http://www.jcst.org/>. Accessed 21st January, 2017.

7. Intercollegiate Surgical Curriculum Programme [www.iscp.ac.uk](http://www.iscp.ac.uk). Accessed 16th May, 2016.

8. Modernising Medical Careers Inquiry Report. <http://www.asgbi.org.uk/mmc-consensus/pdfs/MMC_InquiryReport.pdf>. Accessed 9th December, 2016.

9. *A Reference Guide for Postgraduate Specialty Training in the UK (Gold Guide 2016).* Conference of Postgraduate Medical Deans of the United Kingdom 2016.

10. ISCP General Surgery 2013 Syllabus. <https://www.iscp.ac.uk/static/public/syllabus/syllabus_gs_2016.pdf>. Accessed 21st September, 2016.

11. Promoting Excellence: Standards for Medical Education and Training. <https://www.gmc-uk.org/-/media/documents/promoting-excellence-standards-for-medical-education-and-training-0715_pdf-61939165.pdf>. Accessed 13th June, 2018.

12. Joint Committee for Surgical Training Certification Guidelines for General Surgery. 2016; <https://www.jcst.org/quality-assurance/certification-guidelines-and-checklists/>. Accessed 10th January, 2016.

13. eLogbook supervision codes PDF. <http://e1v1m1.co.uk/wp-content/uploads/2013/11/Supervision-codes-help-guide.pdf>. Accessed 10th January, 2016.

14. eLogbook homepage. <http://www.elogbook.org>. Accessed 3rd December, 2015.

15. Allum W, Hornby S, Khera G, Fitzgerald E, Griffiths G. General Surgery Logbook Survey. *The Bulletin of the Royal College of Surgeons of England.* 2013;95(4):1-6.

16. Miller GE. The assessment of clinical skills/competence/performance. *Acad Med.* 1990;65(9):S63-67.

17. Beard JD, Marriott J, Purdie H, Crossley J. Assessing the surgical skills of trainees in the operating theatre: a prospective observational study of the methodology. *Health Technology Assessment (Winchester, England).* 2011;15(1):i-xxi, 1-162.

18. <https://www.iscp.ac.uk/surgical/assessment_overview.aspx>. ISCP Surgical Curriculum: Assessment. Accessed 03/12/15.
